# Fibrotic progression and radiologic correlation in matched lung samples from COVID-19 post-mortems

**DOI:** 10.1007/s00428-020-02934-1

**Published:** 2020-09-28

**Authors:** Emanuela Barisione, Federica Grillo, Lorenzo Ball, Rita Bianchi, Marco Grosso, Patrizia Morbini, Paolo Pelosi, Nicolò Antonino Patroniti, Arduino De Lucia, Giovanni Orengo, Angelo Gratarola, Marta Verda, Giuseppe Cittadini, Luca Mastracci, Roberto Fiocca

**Affiliations:** 1 Interventional Pulmonology Unit, Policlinico San Martino University Hospital, IRCCS for Oncology and Neuroscience, Genova, Italy; 2 Anatomic Pathology Unit, Department of Surgical Science and Integrated Diagnostics (DISC), University of Genova, Genova, Italy; 3 Policlinico San Martino University Hospital, IRCCS for Oncology and Neuroscience, Genova, Italy; 4 Anesthesia and Intensive Care Unit, Department of Surgical Science and Integrated Diagnostics (DISC), University of Genova, Genova, Italy; 5 Unit of Pathology, University of Pavia and Fondazione IRCCS Policlinico San Matteo, Pavia, Italy; 6 Radiology, Department of Health Sciences (DISSAL), University of Genova, Genova, Italy; 7 General Radiology, Policlinico San Martino University Hospital, IRCCS for Oncology and Neuroscience, Genova, Italy

**Keywords:** Diffuse alveolar damage, COVID-19, Lung fibrosis, Radiologic patterns, Transbronchial lung cryobiopsy

## Abstract

Data on the pathology of COVID-19 are scarce; available studies show diffuse alveolar damage; however, there is scarce information on the chronologic evolution of COVID-19 lung lesions. The primary aim of the study is to describe the chronology of lung pathologic changes in COVID-19 by using a post-mortem transbronchial lung cryobiopsy approach. Our secondary aim is to correlate the histologic findings with computed tomography patterns. SARS-CoV-2-positive patients, who died while intubated and mechanically ventilated, were enrolled. The procedure was performed 30 min after death, and all lung lobes sampled. Histopathologic analysis was performed on thirty-nine adequate samples from eight patients: two patients (illness duration < 14 days) showed early/exudative phase diffuse alveolar damage, while the remaining 6 patients (median illness duration—32 days) showed progressive histologic patterns (3 with mid/proliferative phase; 3 with late/fibrotic phase diffuse alveolar damage, one of which with honeycombing). Immunohistochemistry for SARS-CoV-2 nucleocapsid protein was positive predominantly in early-phase lesions. Histologic patterns and tomography categories were correlated: early/exudative phase was associated with ground-glass opacity, mid/proliferative lesions with crazy paving, while late/fibrous phase correlated with the consolidation pattern, more frequently seen in the lower/middle lobes. This study uses an innovative cryobiopsy approach for the post-mortem sampling of lung tissues from COVID-19 patients demonstrating the progression of fibrosis in time and correlation with computed tomography features. These findings may prove to be useful in the correct staging of disease, and this could have implications for treatment and patient follow-up.

## Introduction

In December 2019, a novel human coronavirus, the severe acute respiratory syndrome coronavirus-2 (SARS-CoV-2), was identified in Wuhan, China [1], and, over the course of a few months, the resulting disease, now known as coronavirus disease 2019 (COVID-19), has spread worldwide, causing a global health emergency.

The clinical presentation of COVID-19 ranges from asymptomatic infection to severe respiratory failure, with fever, fatigue and cough occurring in most cases. Rapid progression to severe respiratory distress requiring intubation and mechanical ventilation has been reported from 7 to 12% of patients (variable percentages in different countries) [2–4]. At imaging, bilateral interstitial pneumonia with characteristic ground-glass features is initially seen with associated crazy paving and consolidation patterns [5].

Data on the pathologic characteristics in COVID-19 are scarce, as post-mortems are infrequently carried out in consideration of the infectious hazard [6]. Indeed, complete post-mortems have been strongly discouraged in some nations (e.g. in Italy, as airborne infection isolation autopsy rooms—BSL3—are not available in most hospitals), while other countries have developed guidelines (e.g. UK [7]) with in-depth recommendations and state that it is preferable, though not necessary, to have isolated BLS3 facilities. Presently, few case reports and series have described the lung pathology in cases of COVID-19 with tissue being sampled either from post-mortem needle biopsies [8], from infection unsuspected surgical resection specimens [9], or from complete/in situ autopsies [10–12]. The descriptions available show diffuse alveolar damage (DAD) with exudates and a predominantly lymphocytic inflammatory infiltrate and large atypical pneumocytes. Pathological findings seem to overlap [13] with those seen in other coronavirus outbreaks, namely, in SARS (severe acute respiratory syndrome) [14, 15] and in MERS (Middle East respiratory syndrome) [16].

In our academic hospital, a specialized multidisciplinary team including lung and intensive care physicians, radiologists, as well as specialized pathologists, with particular expertise in transbronchial lung cryobiopsies (TBLCs) has been routinely performing TBLCs for interstitial lung diseases and has collected considerable experience in over 100 procedures [17]. TBLCs are an innovative procedure which permits the bronchoscopic sampling of lung parenchyma using specialized cryoprobes, which, by rapidly freezing the bronchial wall and adjacent lung parenchyma, enable the operator to sample multiple areas of the lung and obtain biopsies of about 5–6 mm in diameter [18].

Considering (1) the need to follow recommendations which discourage complete post-mortems, (2) the available local expertise (both in terms of TBLC procedure and specialized lung pathology and radiology evaluation) and (3) the interest in obtaining histomorphologic information, a cryobiopsy-based limited post-mortem procedure has been implemented since the 10th of April 2020. The primary aim of the present study was to describe the chronology of lung pathological changes in COVID-19 by using post-mortem (pm) TBLC, in mechanically ventilated patients who died in the intensive care unit (ICU). Our secondary aim was to relate the morphologic findings with computed tomography (CT) imaging patterns.

## Materials and methods

### Patient inclusion criteria

ICU patients who died while intubated and mechanically ventilated with interstitial pneumonia on CT scan and positive polymerase chain reaction for SARS-Cov-2 (either on nasopharyngeal swab or broncho-alveolar lavage) were enrolled. Death was ascertained by a continuous 20-minutes flat electrocardiogram; consent was verbally obtained from the next of kin, and procedure was initiated within 30 min. The protocol was approved by the regional ethics committee (reference number: CER Liguria: 144/2020-DB id 10460 on the 06/04/2020).

### Post-mortem trans-bronchial lung cryobiopsy (pmTBLC) procedure

The methodology described for pmTBLC is slightly different compared with in vivo TBLC; though perhaps inappropriate, the term cryobiopsy (and not cryosample as would be more fitting in the deceased) is used throughout as it refers more to the procedure than the sample itself. All patients were already intubated and mechanically ventilated with an endotracheal tube. At death, heparin infusion was immediately stopped, and subsequently a single-use, flexible video-bronchoscope (Ambu®aScope™, Ambu A/S, Ballerup, Denmark) with a 2.6-mm-wide operative channel was introduced. A 1.7-mm-wide cryoprobe (ErbeCryo®, Erbe Elektromedizin GmbH, Tuebingen, Germany) was inserted within the working channel, and the cryoprobe was then frozen for approximately 10–11 s and biopsy performed.

The sampling protocol included 2 biopsies at each site starting from the right lower lobe (RLL) and continuing to the right middle lobe (RML), right upper lobe (RUL), left lower lobe (LLL) and left upper lobe (LUL). A maximum of 10 biopsies were collected and immediately fixed in 10% buffered formalin.

During all procedures, operators wore full protective equipment including whole body suits, protective hoods, moulded protection masks (FFP3), visors, goggles and double-layer gloves. No operator infection occurred.

### Clinical details and imaging

All significant clinical details were collected for each patient including days from symptom onset, days from arrival in emergency, days of ICU from start of mechanical ventilation, symptoms at presentation, known pre-existing conditions (e.g. hypertension, diabetes, lung disease, heart disease), treatment, tracheotomy, known super-infection and clinical cause of death.

All patients had undergone high-resolution multi-detector CT imaging scans (Siemens Definition Flash, 128 slice, Erlangen, Germany). CT scan evaluation was performed by two experienced radiologists (GC and MV), and the identified patterns were placed in the following categories: ground-glass opacity (GGO), crazy paving (CP), consolidation (C) and honeycombing (HC) according to pulmonary lobes [5, 19, 20].

### Processing of pmTBLC samples

All procedures followed strict guidelines to minimize risk of infection. Samples were formalin fixed (10% buffered formalin) for a minimum of 24 h, and then, tissue was routinely processed and paraffin embedded. From the paraffin blocks, 3–4-μm-thick sections were cut and stained with haematoxylin and eosin (H&E). Immunostaining (cytokeratin 7, CD3, CD20, CD31, CD61, CD68, CD163, p40, TTF1 and smooth muscle actin) was performed on the Ventana BenchMark ULTRA (Ventana Roche Diagnostics, Basel, Switzerland) automated immunostainer as per manufacturer instructions. Mallory’s trichrome (for connective tissue) and alcian blue-PAS (for mucins) stain were performed on the BenchMark Special stains Roche automated stainer as per manufacturer instructions. Double immunohistochemical staining was obtained by developing reactions in brown using UltraView Universal DAB Detection Kit and in red using UltraView Universal Alkaline Phosphatase Red Detection Kit, while double CK7/trichrome staining was obtained by firstly performing immunohistochemistry.

### Histopathologic evaluation of pmTBLC samples

Adequacy was evaluated by considering dimensions of the samples (mm on both axes), surface area (mm^2^) and percentage of lung parenchyma present in each biopsy (at least 30%) [21]. Adequate samples were classified as centrally sampled when predominant bronchi with cartilage plates or bronchiolar structures were seen in more than 40% of surface or peripherally sampled when alveoli were present in at least 60% of surface with or without visceral pleura [22].

All samples were evaluated simultaneously by a panel of experienced pathologists. Histologic elementary lesions (pneumocyte loss with discontinuation of alveolar epithelial lining, hyaline membranes, intra-alveolar fibrinous exudate, early fibroblastic interstitial fibrosis, obliteration of the alveolar structure by fibrosis, type 2 pneumocyte hyperplasia and atypia, Mallory-like intracytoplasmic inclusions in type 2 pneumocytes, micro-honeycombing, foci of bronchopneumonia) were searched separately in all available biopsies and scored as 0 absent, 1 mild and 2 marked. Inflammation was also scored as absent (score 0), mild (score 1) or marked (score 2), and immunohistochemistry was used to distinguish T lymphocytes (CD3+), B lymphocytes (CD20+) and alveolar macrophages (CD68+, CD163+). CD61 was used for the detection of platelet microthrombi and megakaryocytes in all adequate samples [23].

On the basis of the overall expression of the above elementary lesions, one of the following histologic patterns for DAD was assigned to each biopsy site: early exudative phase, mid proliferative phase and late/organizing fibrotic phase with or without honeycombing [24]. A comprehensive pattern assignment, for each patient, was based on the predominant lesions observed as a complete evaluation of all available biopsies. We must underline, however, that pattern recognition is not clear-cut and various patterns may be seen at different sites in the same patient. Indeed, such patterns were assigned taking into account the most represented lesions and not only the worst (when minimal). Histologic patterns were also correlated with biopsy site (upper, middle or lower lobes).

Immunohistochemistry for SARS-CoV-2 was performed on all available samples using an antibody directed against the nucleocapsid protein (rabbit monoclonal anti-nucleocapsid protein, Sino Biological Inc., Chesterbrook, PA, USA), and immunoexpression was evaluated with regard to site (bronchial mucosa, pneumocytes, macrophages, hyaline membranes) and intensity of positivity (score 0–2 where 0 is absent, 1 is mild, 2 is intense) [25, 26]. SARS-CoV-2-positive and SARS-CoV-2-negative controls were used.

### Statistical analysis

Histologic patterns were correlated with radiologic categories by Spearman’s Rho analysis using commercial statistical software (Statistical Package for Social Science, SPSS) and expressed as r_s_ where total concordance is 1.

## Results

### Patient characteristics

Eight patients were included in the study between the 16th of April and the 4th of May 2020. Patients’ median age was 76 years (range 47–79 years); 2 were female and 6 were male. Median time of symptom onset was 5 days prior to hospitalization (range 1–7 days), while admission to ICU and mechanical ventilation was on average 8 days after hospitalization (range 1–18 days). Median stay in ICU was 14 days before death (range 1–29 days). Two patients died from coincidental causes: patient 6 died from haemorrhagic shock following surgery; patient 8 died from cardiogenic shock due to acute myocardial infarction. Patient characteristics are shown in Table 1. All patients, except for patients 1 and 6, had been on non-invasive continuous positive airway pressure before intubation, and all received anti-coagulation treatment and systemic antibiotics/antimycotics.

**Table 1:** Clinical characteristics of patients with fatal COVID-19

Patient	Gender (male/female)	Age (years)	Clinical cause of death	Symptoms	Days from symptom onset to death	Days from hospitalization to death	Days from ICU admission to death	Pre-existing conditions	Specific treatment
1	M	75	Refractory hypoxemia	Fever, tachypnoea	33	26	17	HPT, ischemic heart disease	Tocilizumab, hydroxychloroquine, methylprednisolone
2	F	76	Massive pulmonary embolism	Fever, dyspnoea	24	17	9	None	Tocilizumab, hydroxychloroquine, methylprednisolone
3	M	78	Cardiogenic shock	Fever, dry cough	42	35	17	HPT	Darunavir/ritonavir, hydroxychloroquine, oseltamivir, tocilizumab, methylprednisolone
4	M	59	Refractory hypoxemia	Fever, lipothymia	31	30	29	DM (type II)	Darunavir/ritonavir, hydroxychloroquine, methylprednisolone
5	M	79	Refractory hypoxemia	Fever, dyspnoea	20	16	11	HPT	Hydroxychloroquine, methylprednisolone
6	F	77	Haemorrhagic shock	Asymptomatic	5*	4	4	HPT	None
7	M	69	Refractory hypoxemia	Fever, dry cough	38	33	19	None	Darunavir/ritonavir, hydroxychloroquine, methylprednisolone
8	M	47	Cardiogenic shock	Fever, dyspnoea	14	9	1	HPT, ischemic heart disease, kidney transplant	Methylprednisolone

*ICU* intensive care unit, *HPT* hypertension, *DM* diabetes mellitus

*Patient was asymptomatic and time refers from positive viral PCR

### Biopsy adequacy

Comprehensively, 63 pmTBLCs (median eight per patient, range 6–10) were collected from eight patients; the mean diameter was 6.8 mm in maximum axis and 4.25 in minimum axis and mean surface area was 29.5 mm^2^. Thirty-nine samples were considered adequate, and for each sample site, one or two samples were available for evaluation depending on adequacy. A rather gradual learning curve was necessary to reach a sufficient number of adequate samples (for the first 4 patients, a median of 7 biopsies, of which only a median of 2 adequate biopsies were obtained, while for the following 4 patients, a median of 9 biopsies of which a median of 8 adequate biopsies were obtained) as the technique is slightly different in this setting compared with living patients. Indeed, the fact that patients were intubated and mechanically ventilated as well as the unavailability of a fluoroscope for guidance in ICU required an initial modification of the pmTBLC approach. Of the adequate biopsies, 7 were considered centrally sampled while 32 were peripherally sampled.

### Lung histology on pmTBLC

Elementary lesions for each patient were evaluated and findings are summarized in Table 2. Other, less frequent, histologic lesions were squamous metaplasia and alveolar bronchiolization, only seen in patient 1.

**Table 2:** Summary of main elementary histologic lesions and histologic patterns by biopsy site, as well as CT findings. Lesions are graded as (-) absent, (1) mild and (2) marked

Patient	Biopsy site*	HISTOLOGIC CHARACTERISTICS							Histologic DAD phase per site	CT pattern	Days from CT to pmTBLC	Comprehensive histologic DAD phase
		Hyaline membranes	Intra-alveolar fibrinous exudates	Early fibroblastic interstitial fibrosis	Alveolar obliteration by fibrosis	Micro-honeycombs	Pneumocyte hyperplasia and atypia	Pneumocyte eosinophilic inclusions				
1	RLL	-	-	-	2	2	2	2	Micro-honeycomb	HC	5	Micro-honeycomb
	RML	-	-	-	2	2	2	2	Micro-honeycomb	HC		
	LLL	-	-	-	2	-	2	2	Late phase	C		
2	RML	-	-	1	2	-	2	1	Late phase	GGO	3	Mid-phase
	LLL	-	1	2	-	-	1	1	Mid-phase	CP		
	LUL	2	2	1	-	-	1	-	Mid-phase	GGO		
3	RLL	-	-	2	1	-	1	1	Late phase	C	6	Mid-phase
	LLL	2	2	2	-	-	1	2	Mid-phase	CP		
	LUL	1	2	1	-	-	1	-	Mid-phase	CP		
4	RLL	-	1	2	2	-	2	1	Late phase	C	10	Late phase
5	RLL	-	1	2	2	-	2	1	Late phase	C	7	Late phase
	RML	1	1	2	-	-	2	1	Mid-phase	GGO		
	LLL	-	1	2	2	-	2	-	Late phase	C		
	LUL	-	1	2	2	-	2	1	Late phase	GGO		
6	RLL	-	-	-	-	-	-	-	Normal	N	3	Early phase
	RML	-	-	-	-	-	-	-	Normal	N		
	RUL	2	-	-	-	-	-	-	Early phase	GGO		
	LLL	-	-	-	-	-	-	-	Normal	N		
	LUL	2	1	-	-	-	1	-	Early phase	GGO		
7	RLL	1	2	2	-	-	2	1	Mid-phase	C	10	Mid-phase
	RML	-	2	2	-	-	2	1	Mid-phase	CP		
	RUL	-	2	2	-	-	2	1	Mid-phase	CP		
	LLL	-	2	2	-	-	2	1	Mid-phase	C		
	LUL	-	2	2	1	-	2	1	Mid-phase	CP		
8	RLL	-	-	-	-	-	-	-	Normal	N	10	Early phase
	RML	2	-	-	-	-	-	-	Early phase	N		
	RUL	2	-	1	-	-	1	-	Early phase	GGO		
	LLL	1	1	-	-	-	-	-	Early phase	N		
	LUL	2	1	-	-	-	-	-	Early phase	N		

*RLL* right lower lobe, *RML* right middle lobe, *RUL* right upper lobe, *LLL* left lower lobe, *LUL* left upper lobe, *DAD* diffuse alveolar damage, *CT* computed tomography, *HC* honeycomb pattern, *C* consolidation pattern, *CP* crazy paving pattern, *GGO* ground-glass opacity pattern, *N* normal

*For each biopsy site, one or two biopsies were available for evaluation depending on adequacy, for a total of 39 samples from 29 biopsy sites

**Fig. 1:**
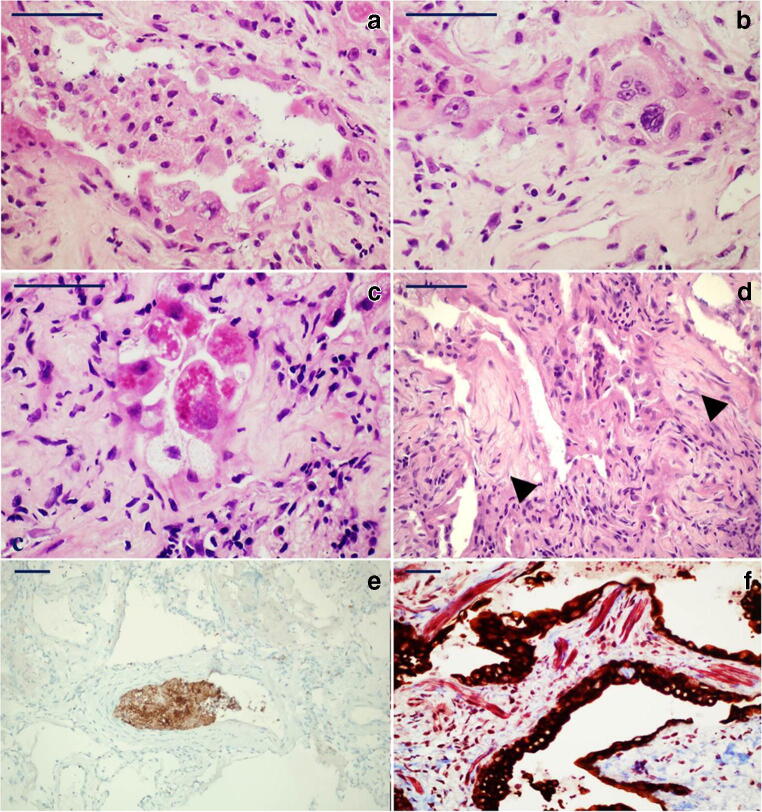
*Elementary lesions commonly seen in the organizing phase of DAD*: **a** Hyperplastic type II pneumocytes line an alveolus inside which an aggregate of macrophages can be seen (H&E, × 60; scale bar 50 μm); **b** atypical pneumocytes with occasional multi-nucleation line a residual alveolar lumen surrounded by early fibrosis (H&E, × 60; scale bar 50 μm). **c** Intracytoplasmic eosinophilic Mallory-like inclusions in type 2 pneumocytes covering alveolar residues surrounded by fibroblastic fibrosis (H&E, × 60; scale bar 50 μm). **d** Fibroblastic foci (arrowheads) are characteristic of the late/organizing phase of DAD and associated with complete derangement/obliteration of alveolar structure (H&E, × 40; scale bar 50 μm). **e** CD61 immuno-stained section showing a platelet microthrombus in a small-sized vessel (CD61, × 20; scale bar 50 μm). **f** Interstitial proliferation of smooth muscle fibres (red) in a micro-honeycombing area; CK7 highlights the bronchiolization of the alveolar epithelium (CK7 immunoperoxidase with Mallory trichrome counterstain, × 20; scale bar 50 μm)

Common features were type II pneumocyte hyperplasia, atypical pneumocytes with occasional multi-nucleation and intracytoplasmic eosinophilic Mallory-like inclusions in type 2 pneumocytes suggestive of proteasome dysfunction (Fig. 1).

Inflammation was generally mild and predominantly composed of macrophages and CD3+ T lymphocytes, while B cells were only observed in sparse aggregates. Macrophages (CD163+, CD68+) were the main inflammatory cell type often associated with intra-alveolar fibrinous exudates. Neutrophilic infiltrates were mild, except for patient 7 who showed diffuse bronchopneumonic neutrophilic inflammation and tested positive for *Pseudomonas aeruginosa* at broncho-alveolar lavage.

With regard to vascular injury, platelet microthrombi were identified using CD61 in 4 patients (patient 2, 6, 7 and 8) both as small capillary thrombi and as one organized thrombus in a small vessel (Fig. 1). Following the report of endotheliitis present in COVID-19 patients [27, 28], this feature was also actively looked for but to no avail.

**Fig. 2:**
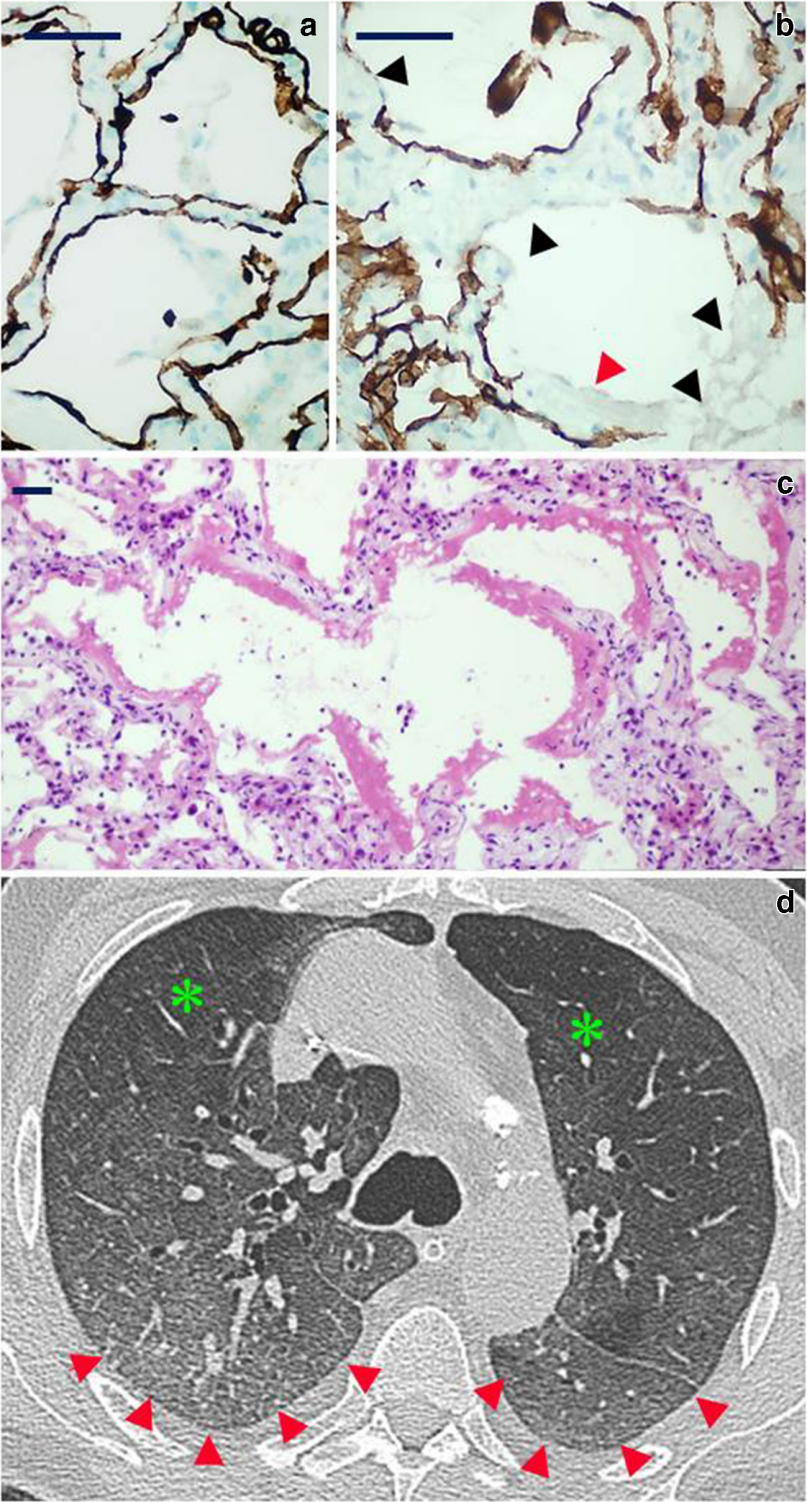
*Histology and radiology of early diffuse alveolar damage (DAD) pattern*: **a** CK7 highlights the continuity of the alveolar epithelial lining in an unaffected area (CK7 immunoperoxidase, × 60; scale bar 30 μm). **b** Loss of the alveolar epithelium (black arrowheads) is accompanied by the appearance of hyaline membranes (red arrowhead) (CK7 immunoperoxidase, × 60; scale bar 30 μm). **c** Intra-alveolar hyaline membranes in the absence of interstitial fibrosis and alveolar distortion (H&E, × 20; scale bar 50 μm). **d** CT findings of diffuse bilateral ground-glass opacities (red arrowheads) defined as hazy, increased opacity of lung, with preservation of bronchial and vascular margins. Normal lung for comparison is identified by green asterisk

The following comprehensive histologic diagnostic patterns (see table 2) could be distinguished:

*Early exudative DAD phase* in two patients—patients 6 (5 days from positive PCR, though asymptomatic) and 8 (14 days of duration from symptom onset): this pattern of injury was characterized by alveolar pneumocyte denudation with intra-alveolar hyaline membrane formation in the absence of interstitial fibrosis or alveolar structure obliteration (Fig. 2). Patient 6 died while asymptomatic but with SARS-*CoV*-2 infection detected on preoperative swab (surgery was for acute gastric haemorrhage due to invasive cancer), showing just focal presence of hyaline membranes in the upper lobe biopsies. The upper and middle lobe biopsies of patient 8 also showed similar changes.

**Fig. 3:**
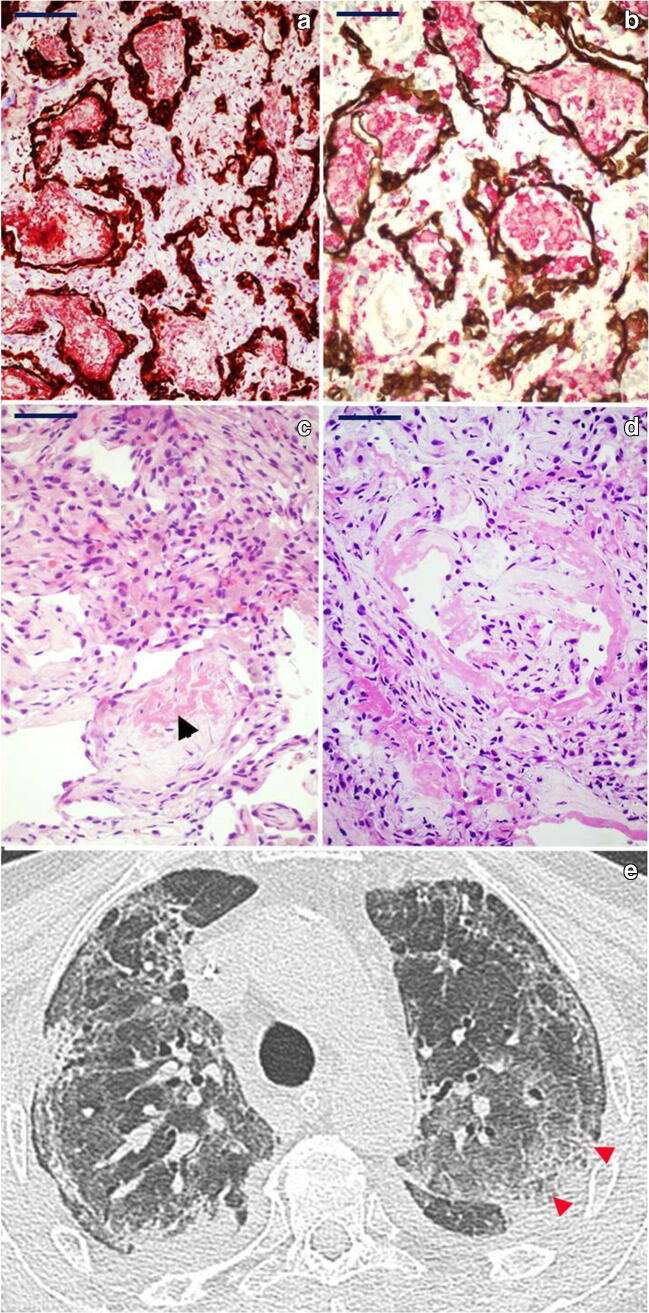
*Histology and radiology of mid-phase DAD pattern*: **a** CK7 and Mallory trichrome, respectively, highlight marked intra-alveolar fibrinous exudates (red) and hyperplasia of the epithelial lining (brown) (CK7 immunoperoxidase and Mallory trichrome counterstain, × 40; scale bar 50 μm). **b** Numerous intra-alveolar macrophages are stained by CD163 (red), while CK7 highlights the epithelial lining (brown); also note the interstitial thickening (CK7 immunoperoxidase and CD163 alkaline phosphatase red; × 40; scale bar 50 μm). **c** Mural incorporation of the intra-alveolar fibrinous exudate (arrowhead) (H&E, × 40; scale bar 50 μm). **d** Intra-alveolar organization of hyaline membrane residues with organizing pneumonia (OP)–like pattern (H&E, × 40; scale bar 50 μm). **e** CT showing a “crazy paving” pattern (red arrowheads), characterized by thickened interlobular septa and intralobular lines superimposed on a background of GGO resembling irregularly shaped paving stones. Peripheral fibrotic bands and traction bronchiolectasia are also present

Patterns of increasing severity of parenchymal involvement (median day of duration from symptom onset: 32 days, range 20–42 days) were as follows:

*Mid proliferative phase DAD* in 3 patients—patients 2, 3 and 7: the main features consisted in marked intra-alveolar fibrinous exudates, filling and expanding alveoli, frequently associated with numerous intra-alveolar macrophages (Fig. 3). These findings were frequently associated with mild fibroblastic fibrosis, with no evidence of mature collagen deposition. Signs of mural incorporation of the intra-alveolar fibrinous exudate and occasional organizing pneumonia–like fibroblastic plugs filling some alveoli and bronchioles were observed.

**Fig. 4:**
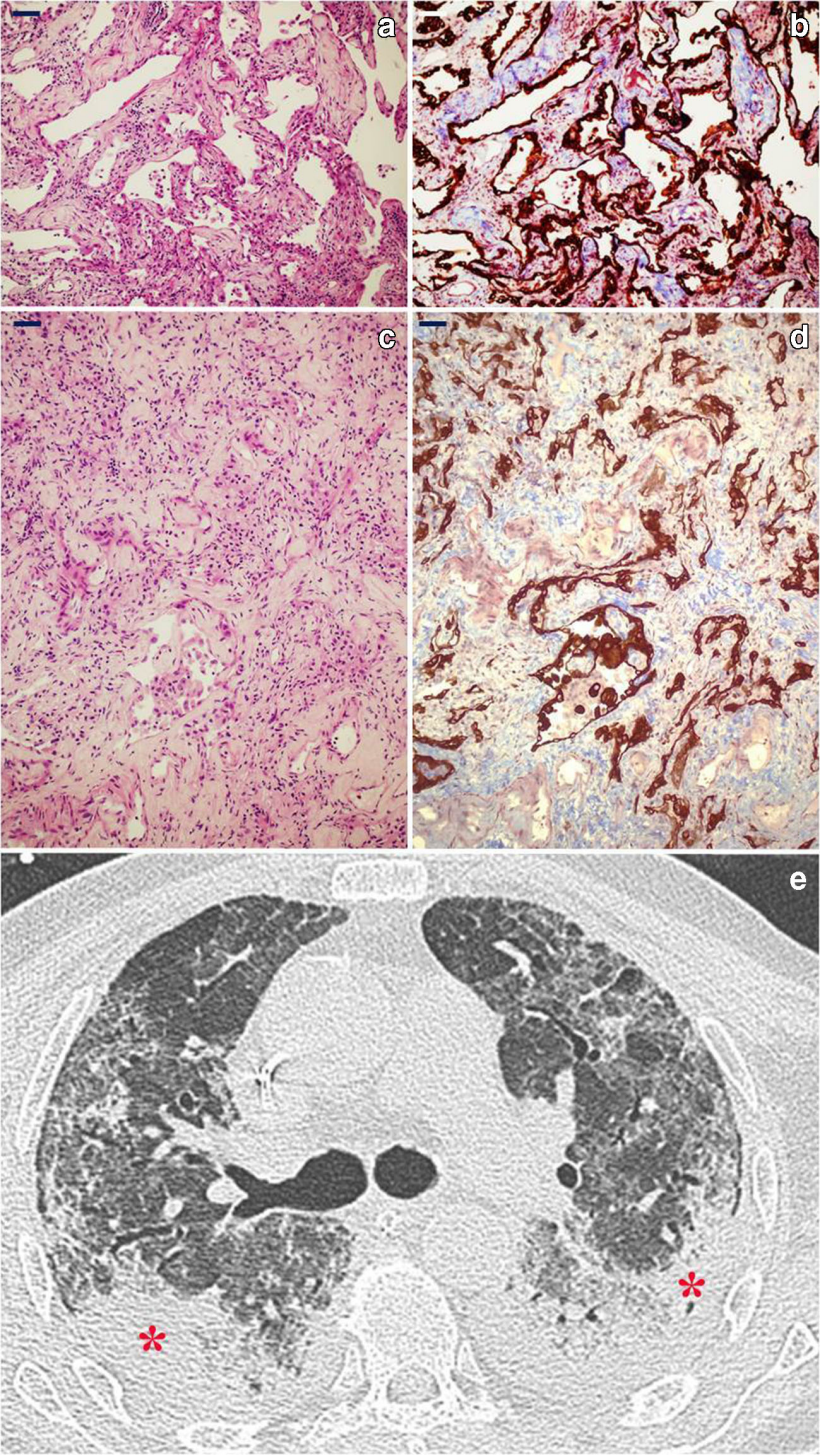
*Histology and radiology of late/organizing phase of DAD pattern*: aspects of progressive derangement/obliteration of alveolar structure by interstitial fibroblast proliferation. **a–b** Alveoli are still recognizable although distorted (**a**, H&E, × 20; **b**, CK7 immunoperoxidase with Mallory trichrome counterstain, × 20; scale bar 50 μm). **c–d** Alveoli become slit-like and compressed by fibrosis and are only readily seen by CK7 immunostaining (**c**, H&E, × 20; **d**, CK7 immunoperoxidase with Mallory trichrome counterstain, × 20; scale bar 50 μm). **e** CT shows advanced parenchymal alterations with predominant dorsal consolidations (homogeneous increase in pulmonary parenchymal attenuation that obscures the margins of vessels and airway walls as opposed to GGO—red asterisks); peripheral crazy paving pattern is seen anteriorly

*Late/organizing fibrotic phase of DAD* in 2 patients—patients 4 and 5: features of organization/repair were characterized by complete derangement/obliteration of alveolar structure by progressive interstitial fibroblast proliferation with sparse collagen fibre deposition. Alveoli were only readily seen by CK7 immunostaining as they become slit-like and compressed by fibrosis (Fig. 4).

**Fig. 5:**
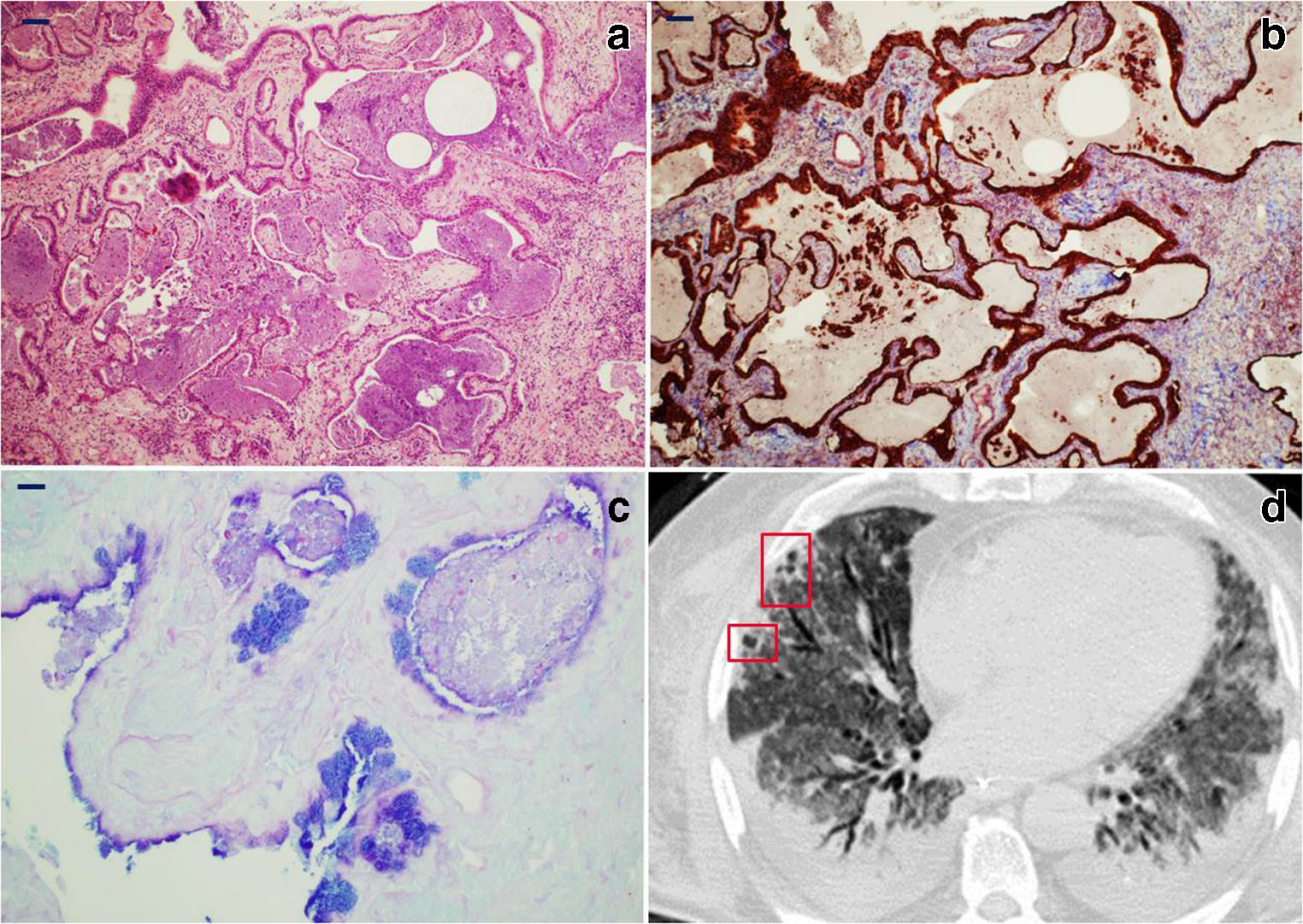
*Histology and radiology of micro-honeycombing pattern*: **a** Complete re-modelling of parenchymal structure by fibrosis and mucinous filling of the alveolar spaces (H&E, × 10; scale bar 100 μm). **b** Mallory trichrome shows collagen deposition (light blue), and CK7 highlights diffuse bronchiolization of epithelial lining (brown) (CK7 immunoperoxidase with Mallory trichrome counterstain, × 10; scale bar 100 μm). **c** Alcian blue/PAS stain highlights diffuse mucinous metaplasia of the epithelial lining (alcian blue/PAS, × 40; scale bar 50 μm). **d** CT shows parenchymal diffuse GGO alterations with initial honeycombing pattern (subpleural clustered cystic air spaces, characterized by well-defined walls) in the middle lobe (red boxes); bilateral pleural effusion and mild consolidations in the posterior lower lobes, with bronchial enlargement, are also seen

*Late/organizing fibrotic phase of DAD with micro-honeycombing* was seen in patient 1 (Fig. 5) characterized by structure re-modelling and bronchiolar metaplasia of re-managed alveoli. Mature collagen, interstitial smooth muscle fibres and squamous metaplasia were also seen in this case.

**Fig. 6:**
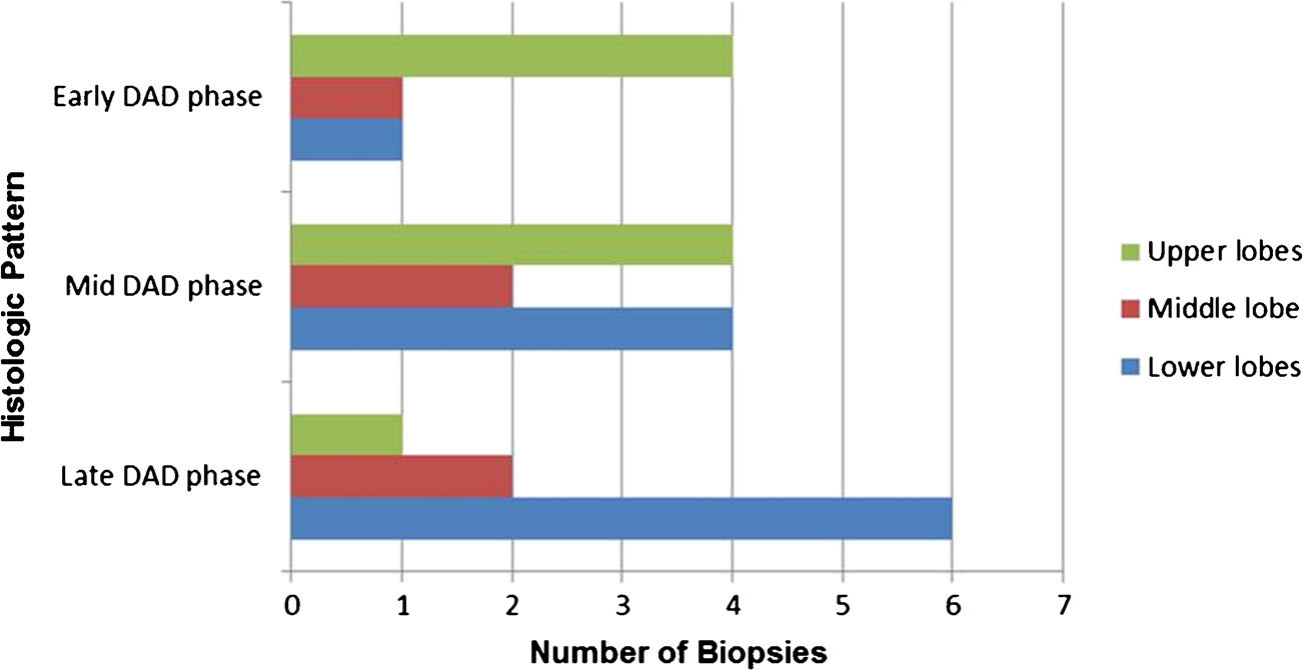
*Histogram showing relation between histologic phase and lung lobe involvement*. The exudative phase is more represented in the upper lobes while the late phase prevails in the lower lobes

Histologic patterns were correlated with biopsy sites showing that early exudative lesions were more frequently found in the upper lobes, while mid- and late-phase patterns were predominantly observed in the lower/middle lobes (Fig. 6).

Pleura with mesothelium was seen in 7 patients (15 samples). Mesothelium appeared either normal or reactive and hyperplastic and the latter were seen in 13 samples from 7 patients, 4 of which also showed inflammation with fibrin deposition.

### Immunohistochemistry for nucleocapsid protein of SARS-CoV-2

**Fig. 7:**
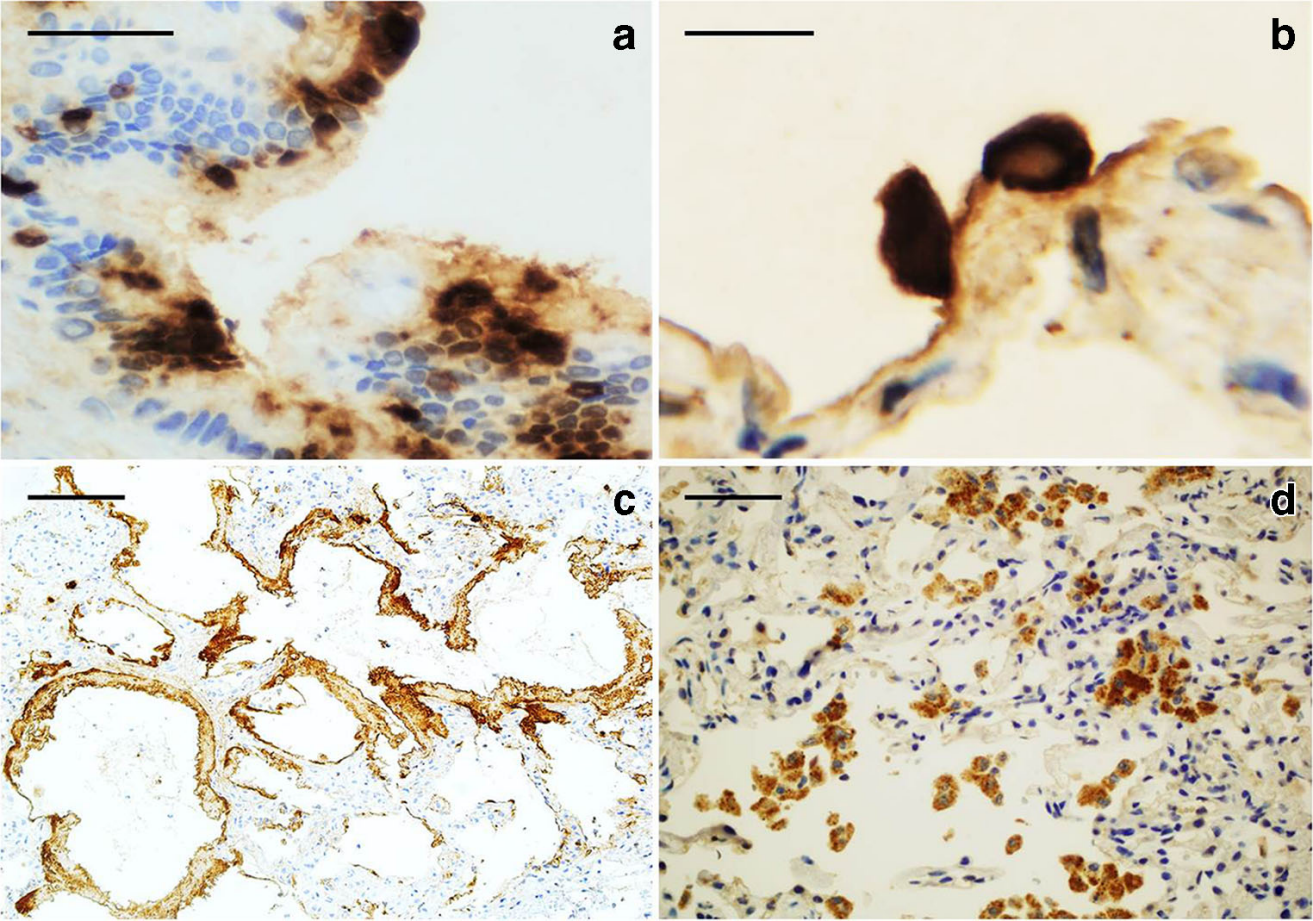
*SARS-CoV-2 nucleocapsid protein detection by immunohistochemistry.* **a** Immunopositivity in the cytoplasm of bronchial cells and as a rim on their luminal border (× 60; scale bar 50 μm). **b** Immunopositivity in the cytoplasm of pneumocytes lining the alveolar space in early exudative phase DAD (× 100; scale bar 25 μm). **c** Immunoreactivity of hyaline membranes in early exudative phase DAD (× 20; scale bar 100 μm). **d)** Immunopositive alveolar macrophages in exudative phase DAD (× 40; scale bar 50 μm)

Positive staining for anti-nucleocapsid SARS-CoV-2 protein was clearly present in patients 6 and 8 with early exudative phase lesions. Staining was diffuse and intensely positive in hyaline membranes, pneumocytes and macrophages as well as scattered positive bronchial cells with a positive cytoplasm and luminal rim (Fig. 7). All other patients, with mid-proliferative and late/organizing fibrotic phase, did not show staining in macrophages or pneumocytes except for faint staining in residual foci of exudative lesions.

### Correlation between lung histologic patterns and radiology findings

CT scans were performed within 5 days prior to death for all patients except for patient 4, patient 7 and patient 8 whose last CT scan was 10 days before death (median between CT and death 6 days, range 1–10) but was re-assessed by chest X-rays within the last 3 days from death.

**Fig. 8:**
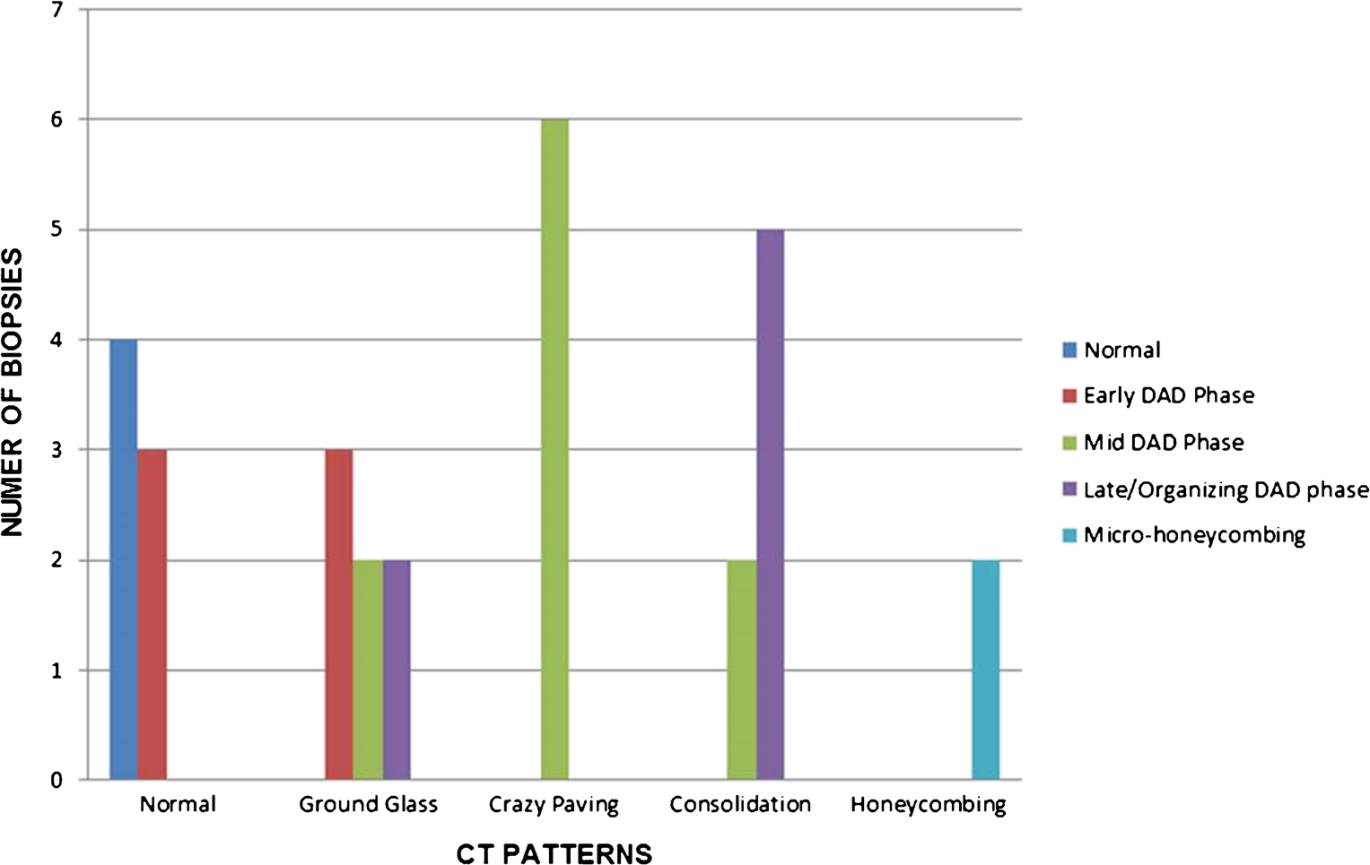
*Correlation between histologic and radiologic CT patterns (see also* Table 3*).* Early damage was found either in apparently healthy lung regions at imaging or associated with ground-glass opacities. Crazy paving was the most characteristic feature of the mid DAD phase, while consolidation and honeycombing were associated with the late-phase histologic changes

As shown in Table 3 and Fig. 8, sites with normal CT findings corresponded to normal histology in 4 sites and early exudative phase DAD in 3 out of 7 sample sites. Sites with GGO at CT showed a mixture of patterns ranging from early to mid to late phases (3/7 versus 2/7 versus 2/7, respectively), while areas of CP all corresponded to mid proliferative phase histologic patterns (6 sample sites). Finally consolidation at CT correlated with 2 mid-phase and 5 late/organizing-phase sites out of 7. The only 2 biopsy sites with micro-honeycombing at histology came from the same patient who showed marked consolidation, calcifications and honeycombing at imaging. Comprehensively, histology patterns and CT categories showed significant correlation (r_s_ = 0·84) in the 29 biopsy sites evaluated.

**Table 3:** Correlation between histologic patterns and CT features for each biopsy site (Spearman’s Rho: R_s_ 0·8)

		CT features				
		Normal	Ground glass	Crazy paving	Consolidation	Honeycombing
Histologic pattern	Normal	Patient 6—RLL/RML/LLL Patient 8—RLL				
	Early-phase DAD	Patient 8—RML/LLL/LUL	Patient 6—RUL/LUL Patient 8—RUL			
	Mid-phase DAD		Patient 2—LUL Patient 5—RML	Patient 2—LLL Patient 3—LLL/LUL Patient 7—RML/RUL/LUL	Patient 7—RLL/LLL	
	Late/organizing-phase DAD		Patient 2—RML Patient 5—LUL		Patient 1—LLL Patient 3—RLL Patient 4—RLL Patient 5—RLL/LLL	
	Micro-honeycombing					Patient 1—RLL/RML

*RLL* right lower lobe, *RML* right middle lobe, *RUL* right upper lobe, *LLL* left lower lobe, *LUL* left upper lobe, *DAD* diffuse alveolar damage

*For each biopsy site, one or two biopsies were available for evaluation depending on adequacy, for a total of 39 samples from 29 biopsy sites

## Discussion

In this study, the post-mortem transbronchial lung cryobiopsy technique permitted sampling of different lung lobes in 8 mechanically ventilated SARS-CoV-2-positive patients. The main findings of this study include the following: (1) the identification of a chronological evolution of lesions from an early exudative phase with hyaline membranes to a mid-phase characterized by intra-alveolar fibrinous exudate and early fibroblastic interstitial fibrosis to a late phase with alveolar obliteration by fibrosis and possible micro-honeycombing; (2) mild degree of inflammatory infiltrates; and (3) correlation of histologic patterns with lung CT alterations.

To our knowledge, this is the first study which uses the innovative approach of post-mortem transbronchial lung cryobiopsy for tissue collection. Though sampling bias should definitely be kept in mind, the cryobiopsy technique permits relatively large biopsy samples (mean of nearly 30 mm^2^) obtained from ventilated lungs just 30 min after death with nearly no crush artefacts on fresh samples [21] in a protected environment for the operator. A further advantage is that the operator identifies precisely the bronchus from which the surrounding lung parenchyma is obtained making radiological correlation extremely accurate. Similar comparable histologic findings are absent in living patients, making it impossible to understand in vivo pathological evolution.

Most of our patients showed variable severity of fibrosis, from early interstitial to severe obliteration of alveolar structure with organizing predominant features. Previous histologic reports have described bilateral DAD with intra-alveolar exudates, hyaline membrane formation, pneumocyte hyperplasia/atypia and loss with desquamation, as well as other early changes such as alveolar oedema and proteinaceous exudates, with major inflammatory infiltration [8, 9, 12], possible consolidation with fibroblastic proliferation [10, 29], and/or pulmonary thromboemboli [11, 30]. The increased fibrosis, reported in our patients compared with the previous studies, may be explained by different factors including general medical treatment, by the duration of mechanical ventilation or by the evolution of disease towards fibrotic phases. In our study, the duration of symptoms before death was longer than 20 days in all but two patients, with a maximum of 42 days, compared with other studies in which mean length of disease was just 5 to 7 days [10, 12]. Our data suggest that patients with longer spans of illness exhibit more interstitial fibrosis than those with shorter disease course. Furthermore, fibrosis was found even though all patients received anti-inflammatory treatment during the course of the disease. In particular, regarding patient 1who showed a late/organizing fibrotic phase of DAD with micro-honeycombing, the parenchymal re-modelling finds no justification in previous pulmonary disease, as shown by a total body CT scan performed in 2013 following an accident, which revealed normal lungs; furthermore, the patient referred no respiratory symptoms whatsoever till the onset of COVID-19.

Immunohistochemistry for SARS-CoV-2 nucleocapsid protein also showed modification during disease progression as intense immunostaining was seen in early exudative phase lesions, while little or no immunostaining was identified in proliferative and late/organizing fibrotic phases, similarly to findings in two recent studies [23, 31].

Much importance has been placed on vascular injury, endotheliitis and the hyper-coagulative state which seem to be important aspects of COVID-19 [11]. We identified platelet microthrombi (CD61+) in 4 patients to support this; patient 2 was also shown by imaging (echocardiogram positive) to have massive pulmonary thromboembolism. No large vessel thrombi, as identified in full post-mortems, were seen, probably due to sampling variability and/or the peripheral location of pmTBLCs.

A crucial aspect, which may have far-reaching therapeutic implications, is the inflammatory infiltrate in the lung parenchyma of COVID-19 patients. We have seen that macrophage accumulation is evident only in the early/mid-phases of disease, while numbers dwindle with increasing fibrosis. Macrophages, part of the innate immune response, are known to eliminate infectious agents and promote tissue repair through cytokine release. Interestingly, as suggested by Merad and Martin [32], subsets of macrophages in COVID-19 may express activated genes associated with tissue repair and this would explain the evolution to fibrosis which we have observed in our patients. It would prove useful, but difficult, to closely correlate disease timeline and extent of fibrosis, considering the many variables involved, including viral load, cytokine response and patient’s variability.

CT scan findings have been extensively documented and prove useful for both diagnosing and following progression in COVID-19 lung manifestations [33]. Histology correlates with various imaging patterns. Briefly summarizing, we found that crazy paving pattern at CT was associated with mid-phase DAD while the consolidation CT pattern was mainly correlated with late-phase DAD. Ground-glass opacities may be seen isolated in the early phases or as part of a more complex pattern in later progressive disease. Any discrepancies between histologic and CT findings may be partly explained by lapse between CT scan and death (in some cases 10 days) or superimposed bronchopneumonia (patient 7 showed mid-phase DAD but had consolidated CT findings as a possible consequence of superimposed, diffuse, histologically proven, bronchopneumonia).

We may expect that an, as yet, unknown number of COVID-19 survivors who required mechanical ventilation for a long period of time might present lung function impairment with unresolved fibrotic interstitial lung disease [34]. SARS epidemic data show that, within the first year from acute illness, a relevant rate of survivors (15–20%) showed reduced lung functional capacity [35]. Furthermore, a recent study with long-term follow-up demonstrated that, in patients with severe acute SARS related illness, lesions rapidly improved within the first year and then plateaued in the following 15 years with chronic CT lesions and lung function impairment [36]. In consideration of this, a complete and long-term follow-up, with functional studies, imaging and even cryobiopsies, could be necessary for COVID-19 patient management.

Limitations of this study which need to be addressed are the possibility of sampling variability and the inadequacy of many of the first samples due to the necessary learning curve. With regard to the former, biopsy sampling is clearly more limited if compared with full lung post-mortem evaluation, which collects greater information on the spatial distribution of lesions, and therefore, this should be taken into account. However, considering that TBLC are routinely performed for the diagnosis of interstitial lung disease, this itself validates the procedure, even with limitations. A further limitation of the study is the small number of procedures performed in a cohort of patients with a variety of demographic and clinical characteristics, as well as variable medical treatment.

In conclusion, COVID-19 lung injury is characterized by a diffuse alveolar damage histologic pattern with possible major fibrotic evolution in lungs of patients dying after a long period of mechanical ventilation. The knowledge of the histologic features which accompany the different phases of disease, and in particular with regard to fibrous progression, as well as the correlation between histology and CT patterns, constitutes a useful basis for interpreting the meaning of lesions that we may encounter in COVID-19 survivors as well as being the rational basis for the development of new treatments for which an accurate timing of lesions may be necessary.

## Data Availability

Data and material are available in local databases, upon request.
